# Supplementary figures and images for: Angiogenic Function of Human Placental Endothelial Cells in Severe Fetal Growth Restriction Is Not Rescued by Individual Extracellular Matrix Proteins
Source: Cells. 2023 Sep 23;12(19):2339. doi: 10.3390/cells12192339 (PMC10572031; doi:10.3390/cells12192339)

## Slide 1
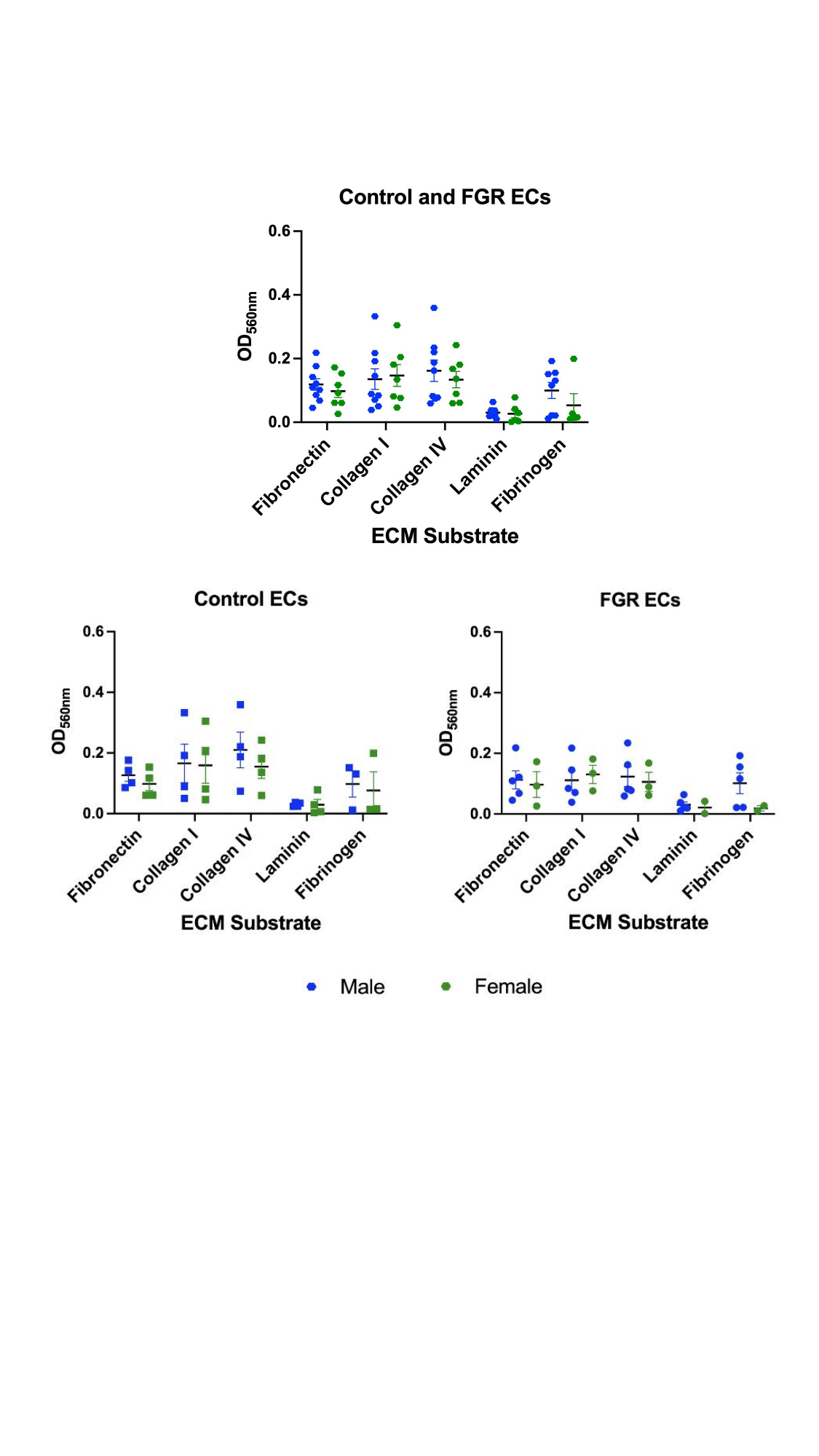

Supplement: Supplementary file 1 [file cells-12-02339-s001.zip › Supplementary Files/Supplemental Figure 1.pptx]

## Slide 1
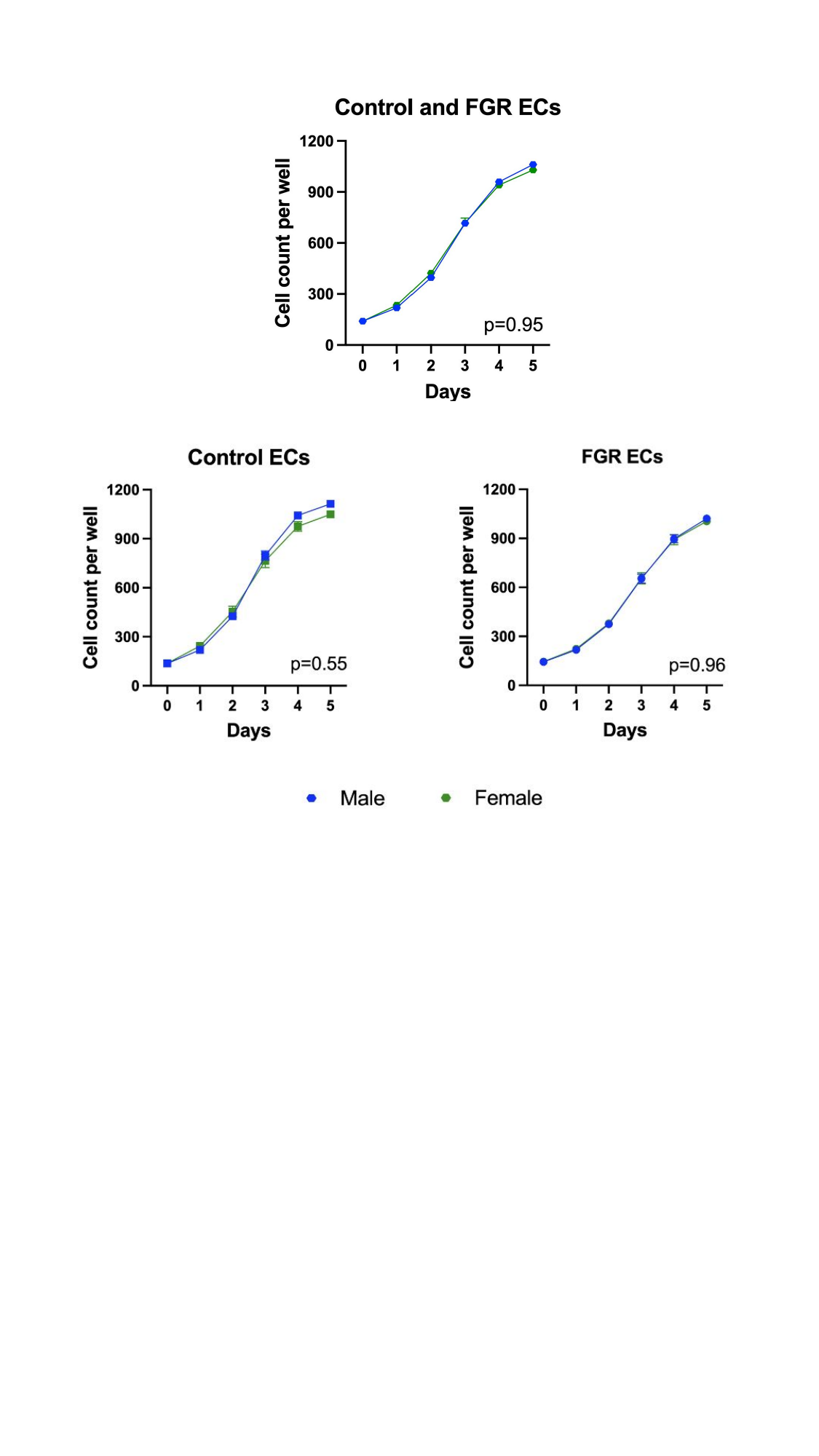

Supplement: Supplementary file 1 [file cells-12-02339-s001.zip › Supplementary Files/Supplemental Figure 2.pptx]

## Slide 1
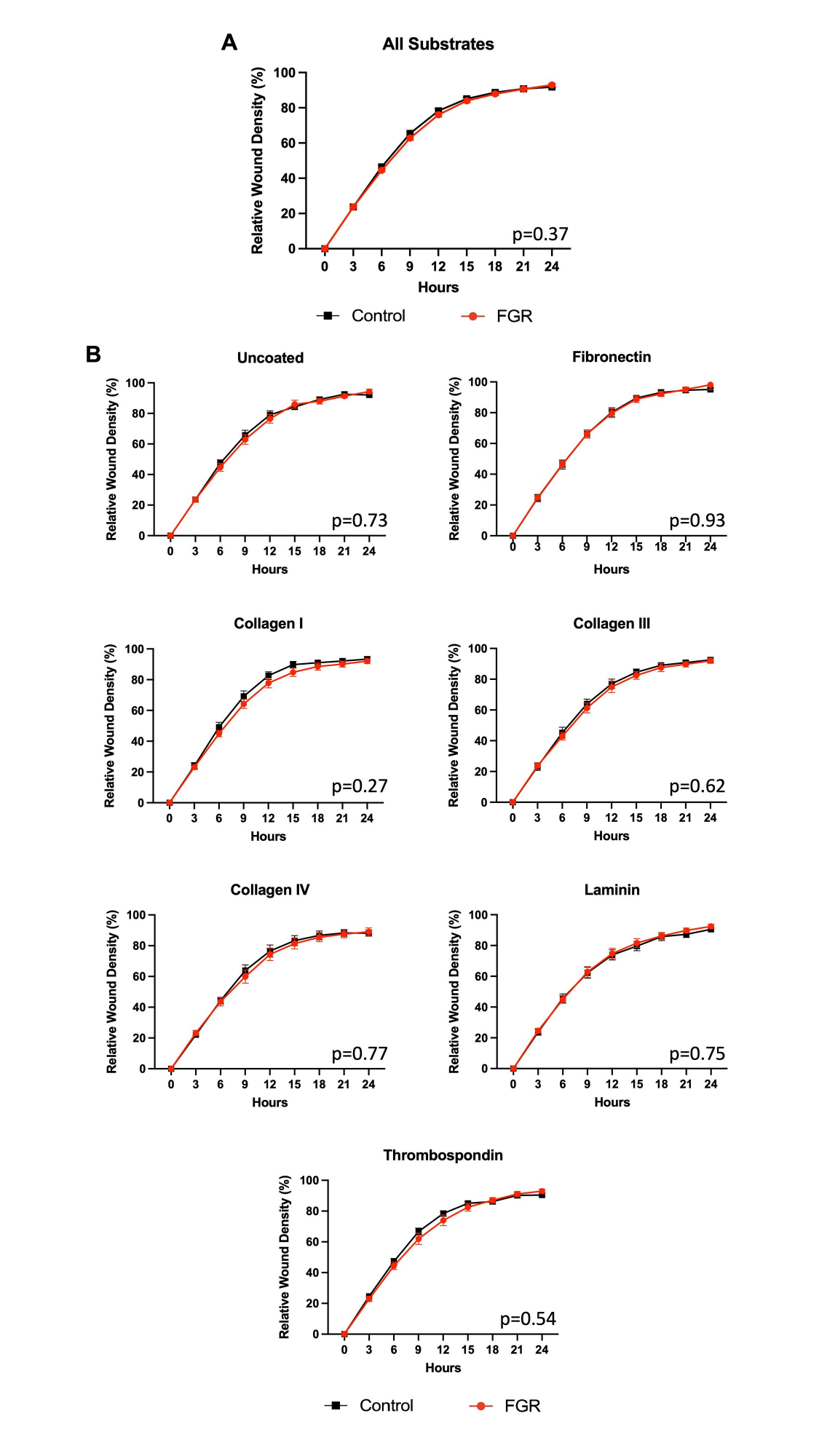

Supplement: Supplementary file 1 [file cells-12-02339-s001.zip › Supplementary Files/Supplemental Figure 5.pptx]

## Slide 1
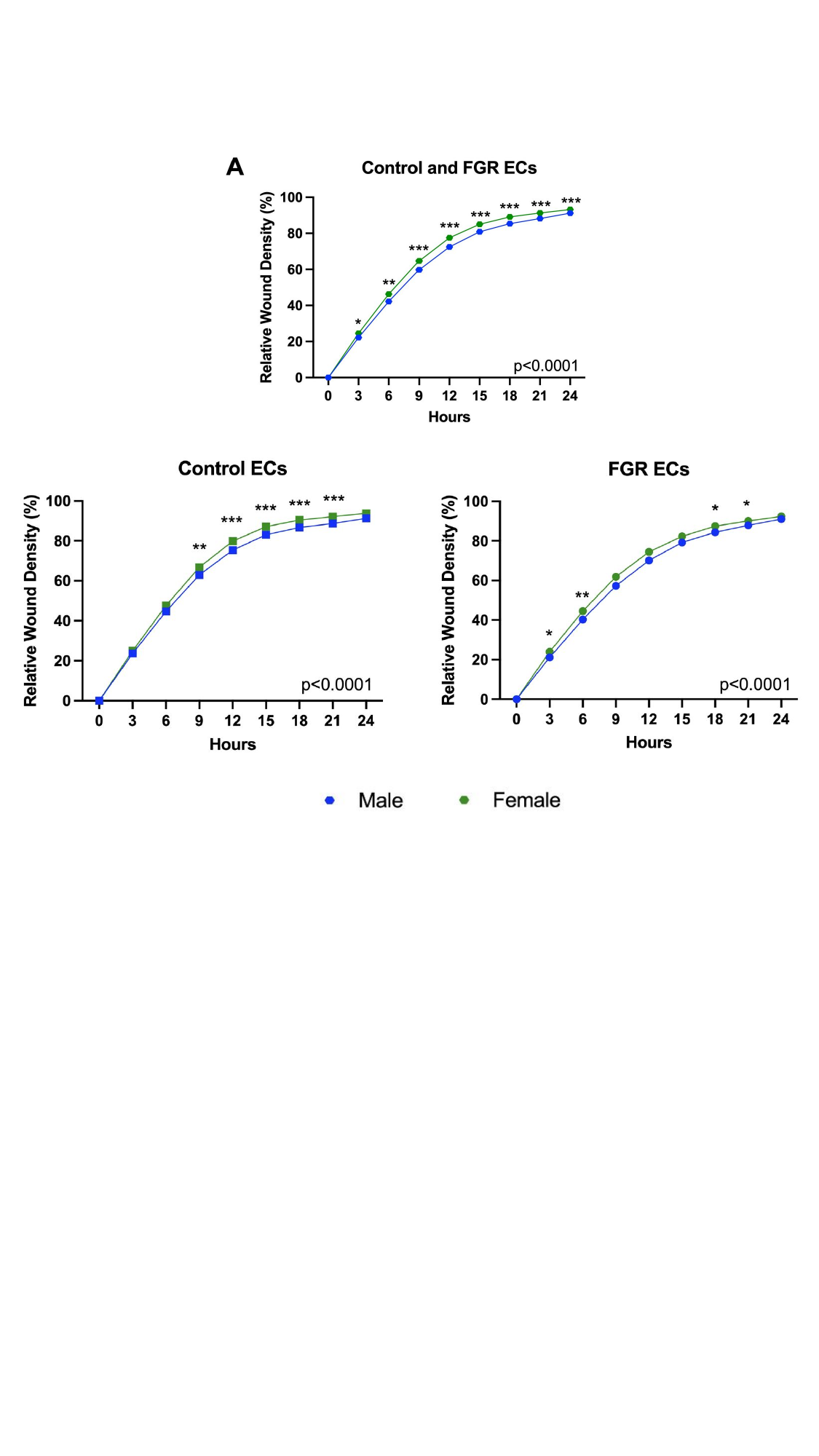

Supplement: Supplementary file 1 [file cells-12-02339-s001.zip › Supplementary Files/Supplemental Figure 4.pptx]
